# Voting, health and interventions in healthcare settings: a scoping review

**DOI:** 10.1186/s40985-020-00133-6

**Published:** 2020-07-01

**Authors:** Chloe L. Brown, Danyaal Raza, Andrew D. Pinto

**Affiliations:** 1 Faculty of Medicine, University of Toronto, Toronto, ON Canada; 2 Department of Family and Community Medicine, St. Michael’s Hospital, Toronto, ON Canada; 3 Department of Family and Community Medicine, Faculty of Medicine, University of Toronto, Toronto, ON Canada; 4 Upstream Lab, MAP/Centre for Urban Health Solutions, Li Ka Shing Knowledge Institute, St. Michael’s Hospital, Toronto, ON Canada; 5 Institute for Health Policy, Management and Evaluation and the Division of Clinical Public Health, Dalla Lana School of Public Health, Toronto, ON Canada

**Keywords:** Voting, Political participation, Democratic engagement, Self-rated health, Health inequities, Social determinants of health

## Abstract

**Background:**

In democracies, voting is an important action through which citizens engage in the political process. Although elections are only one aspect of political engagement, voting sends a signal of support or dissent for policies that ultimately shape the social determinants of health. Social determinants subsequently influence who votes and who does not. Our objective is to examine the existing research on voting and health and on interventions to increase voter participation through healthcare organizations.

**Methods:**

We conducted a scoping review to examine the existing research on voting, health, and interventions to increase voter participation through healthcare organizations. We carried out a search of the indexed, peer-reviewed literature using Ovid MEDLINE (1946–present), PsychINFO (1806–present), Ebsco CINAHL, Embase (1947–present), Web of Science, ProQuest Sociological Abstracts, and Worldwide Political Science Abstracts. We limited our search to articles published in English. Titles and abstracts were reviewed, followed by a full-text review of eligible articles and data extraction. Articles were required to focus on the connection between voting and health, or report on interventions that occurred within healthcare organizations that aimed to improve voter engagement.

**Results:**

Our search identified 2041 citations, of which 40 articles met our inclusion criteria. Selected articles dated from 1991–2018 and were conducted primarily in Europe, the USA, and Canada. We identified four interrelated areas explored in the literature: (1) there is a consistency in the association between voting and health; (2) differences in voter participation are associated with health conditions; (3) gaps in voter participation may be associated with electoral outcomes; and (4) interventions in healthcare organizations can increase voter participation.

**Conclusion:**

Voting and health are associated, namely people with worse health tend to be less likely to engage in voting. Differences in voter participation due to social, economic, and health inequities have been shown to have large effects on electoral outcomes. Research gaps were identified in the following areas: long-term effects of voting on health, the effects of other forms of democratic engagement on health, and the broader impact that health providers and organizations can have on voting through interventions in their communities.

## Background

The idea that health is strongly determined by social factors and processes—what we now call the social determinants of health—has long been a central idea within public health [1]. All social determinants of health are shaped by the distribution of power and resources within societies and at a global level [2, 3]. A number of processes influence this distribution of power and resources, including constitutions that define the rights and responsibilities of citizens and governments, policies that determine the minimum wage, work conditions, and social assistance, as well as the budgetary decisions that direct resources toward (or away from) education, child development, housing, and social services.

In democracies, citizens can play a variety of roles in the processes that shape the social determinants. Voting is one key aspect of democratic engagement, defined as “a multi-faceted phenomenon that embraces citizens’ involvement with electoral politics, their participation in ‘conventional’ extra-parliamentary political activity, their satisfaction with democracy and trust in state institutions, and their rejection of the use of violence for political ends” [4]. More simply, democratic engagement is “the state of being engaged in advancing democracy through political institutions, organizations, and activities” [5]. Democratic engagement can include *electoral participation* (voting, campaign displays, volunteer, campaign contributions), expressing a *political voice* (protest, boycott, contacting officials), having *political knowledge/awareness* (following government affairs, watching/reading/listening to news, talking about politics), and holding certain *attitudes* (promoting common good, affirming common humanity) [5].

The effect of voting on the social determinants of health is multi-factorial and complex. In a simple conceptualization, when larger numbers of people from certain communities and groups participate in voting, it translates into greater influence over determining who holds political power. Those in power in turn put forward and support policies that respond to the needs and demands of their constituents that shape the social determinants of their health. Not only does voting partially decide who forms government in democracies, and subsequently what policies shape social determinants, but the relationship may work in the opposite direction as well, in that the social determinants of health affect voting patterns. For example, socioeconomic status is associated with the likelihood of voting. Across many contexts, having a low income and a lower level of education is associated with lower rates of voting during elections [6–8]. Numerous theories explain this association including decreased social trust, diminished social capital, fewer chances to vote, and weakened educational opportunities about the policy process [7, 8].

Public health scholars have been called upon to better understand the functioning of politics at national and sub-national levels—and the mechanisms that connect politics to public health [9]. Our objective in this scoping review was to examine the existing research on voting and health, and on interventions to increase voter participation through healthcare organizations. We sought to understand the following questions: What is the relationship between voting and individual health? What healthcare-based interventions exist to support voting, and what have been their outcomes?

## Methods

We conducted a scoping review with the central objective of identifying the existing peer-reviewed research on the association between voting and health, and on interventions that aim to increase voter participation through healthcare organizations [10, 11]. Voting was used as a proxy for democratic engagement in this scoping review as it is easily identifiable, measurable, and is an essential and defining characteristic of healthy democracies. We chose to focus on healthcare-based interventions, to explore the role that the health sector—which has frequent contact with large numbers of individuals from communities with relatively lower rates of voting—can play in supporting voting. We searched Ovid Medline (1946–present), PsychINFO (1806–present), Ebsco CINAHL, Embase (1947–present), Web of Science, Proquest Sociological Abstracts, and Proquest Worldwide Political Science Abstracts in March 2018. We used a broad search expression (Additional file 1, Additional file 2) in order to include as many articles as possible. Our search timeframes were chosen to include the full scope of articles available on each research platform. We limited our search to the peer-reviewed, indexed literature in English. The titles and abstracts of citations identified were reviewed independently against our inclusion and exclusion criteria by two authors (CB, DR), followed by review of the full-text articles. In this scoping review, we did not perform backward reference tracking.

We included peer-reviewed articles where the main focus was the relationship or association between voting and individual health, or focused on interventions in healthcare settings aimed at increasing voter participation. We excluded articles solely focused on the links between health and other forms of democratic engagement (ex. activism, protest) to focus more narrowly on the link between the act of voting and health.

After our initial review of full-text articles to ensure they met our inclusion and exclusion criteria, two authors (CB, DR) completed data extraction. We extracted information on the geographic location and context, area of focus, the effect size, measures used, and confounding variables. We prepared summaries for each article on the key themes and findings in one shared document, and then the entire study team reviewed these summaries and identified common overarching themes relevant to our review objectives.

## Results

**Fig. 1:**
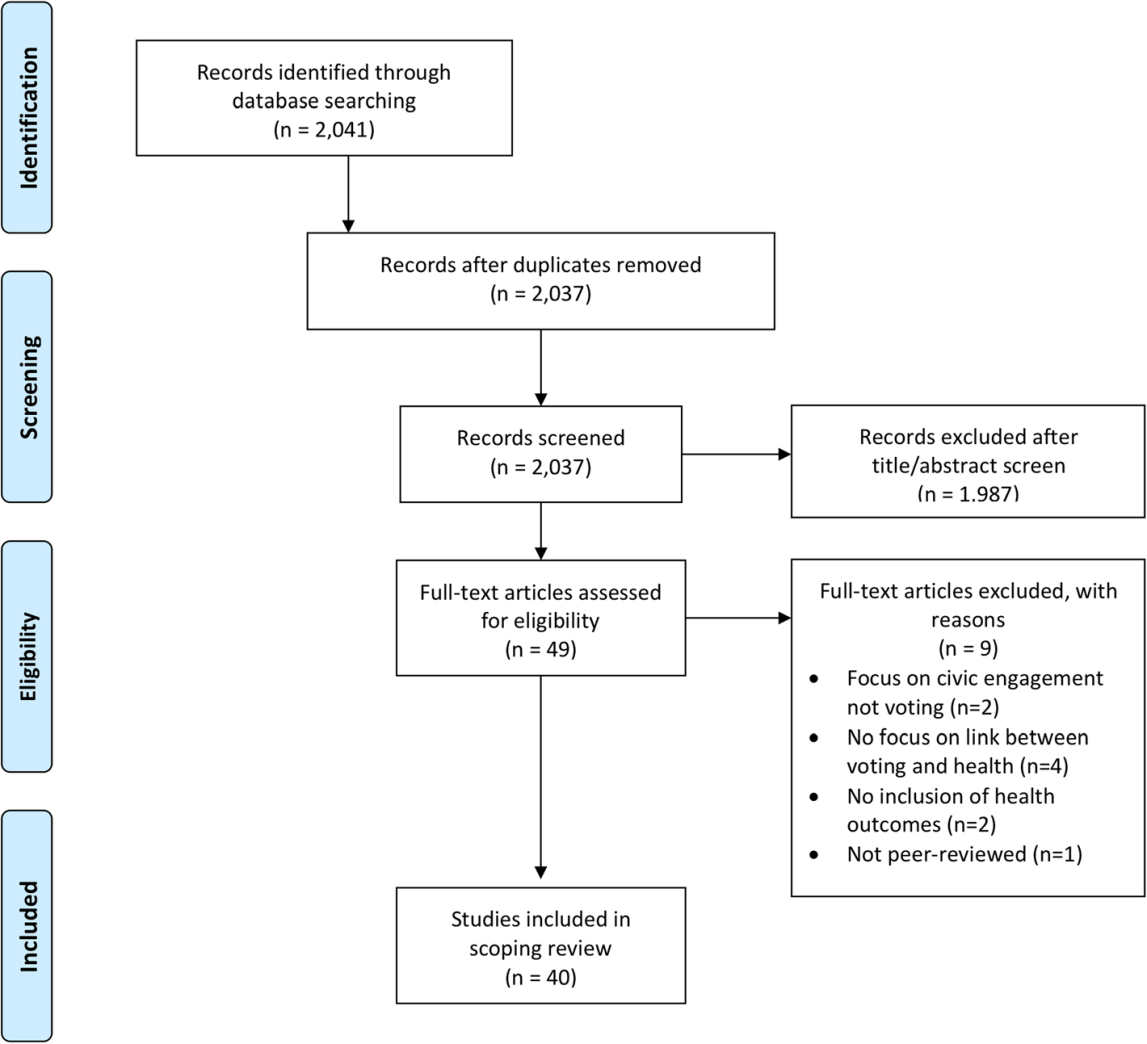
PRISMA flow diagram

Our initial search identified 2041 citations (Fig. 1), and after reviewing titles and abstracts, 49 articles met our inclusion criteria. Following full-text review, 40 articles were included in the final analysis (Table 1). As we put in place a broad search strategy, many articles were not relevant to the research questions. Most of those were articles that focused on subjects like healthcare policy, democratic engagement (activism, civic engagement), voting patterns, political engagement, and health equity more broadly without actually discussing the link between voting and health or describing healthcare interventions in the voting process. The included articles were published in a diversity of research disciplines (classified according to journal and study design): health science (geriatrics, pediatrics, psychiatry), public health and epidemiology, political science, and social science. Most of the research was done in high-income countries, with a focus on Europe, the USA, and Canada. Although most of the research has been more recent, with 27 articles being written from 2010–present, the articles included were published between 1991 and 2018. Study designs included cross-sectional studies, cohort studies, case studies, qualitative studies, literature reviews, and critical commentaries.

**Table 1:** Articles identified that examine voting, health, and interventions in healthcare settings

Authors (year)	Geographic location	Topic area	Main findings	Measures	Confounders addressed
Albright et al. (2016) [12]	Colorado, USA	Association between health and voting	Negative association between health risk behavior (smoking) and voting in the national US election Daily smokers were 60% less likely to vote than nonsmokers (OR: 0.38, 95% CI: 0.27% to 0.54%).	Self-reported smoking status; self-reported voting in the 2004 national US election	Sex, age, race/ethnicity, education, employment status, marital status, household income relative to the federal poverty level, and self-reported general health status
Arah (2008) [13]	Britain	Association between health and voting	Study demonstrates effect of voting abstention in the UK general election and socioeconomic status on self-reported health Abstaining from voting in 1979, 1981, 1997 and 2001 increased odds of poor health in 1981 (1.56, 95% CI 1.36 to 1. 79), 1991 (1.37 95% CI 1.18 to 1.60), 2000 (1. 45, 95% CI 1.28 to 1.66), and 2004 (1.30, 95% CI 1.11 to 1.51).	Self-reported health; self-reported voting in the UK general election (data from National Child Development Study)	Sex, geographic region, age at leaving education, body mass index, chronic illness, and smoking and alcohol consumption frequencies
Ballard, Hoyt, & Pachucki (2018) [14]	USA	Association between health and voting	Positive association between civic engagement during late adolescence/early adulthood, and socioeconomic status and mental health in adulthood (decreased risky health behaviors (ES = − 0.12, SE = 0.018, *p* < 0.001) and fewer depressive symptoms (ES = −0.056, SE = 0.018, *p* = 0.003)	General health, symptoms, physical limitations, depressions, BMI, physical activity, health risk behaviors; Self-reported voting in the US presidential election (data from National Longitudinal Study of Adolescent to Adult Health)	Demographic characteristics, health variables, social connections
Blakely, Kennedy & Kawachi (2001) [6]	USA	Association between health and voting	Socioeconomic inequality in the US state election voter turnout is associated with poor self-rated health, independent of income inequality and household income. Individuals living in the USA with highest voting inequality had an odds ratio of fair/poor self-rated health of 1.43 (95% confidence interval (CI) = 1.22, 1.68) compared with individuals living in the USA with lowest voting inequality.	Self-reported health; self-reported voting in the US State elections (data from Current Population Survey)	Income and state level inequality; age, sex, race, and equivalized household income at individual level
Burden et al. (2017) [15]	WI, USA	Association between health and voting	Positive association between cognitive functioning and voting, and health functioning and voting in older Wisconsin population Better health boosts likelihood of voting by 5% in the 2008 election to 15% in the 2012 election.	Cognitive functioning, Health Utilities Index (HUI); Catalist voting records for the 2008, 2010, and 2012 US State elections (data from Wisconsin Longitudinal Study)	IQ, age, income, education, gender
Couture and Breux (2017) [16]	Canada	Association between health and voting	Positive association between self-rated health and national electoral participation (statistically significant) Positive association between self-rated mental health and municipal electoral participation (reduction in participation of 9.1% for local elections between the respondents who reported “very good” health and “very bad” health)	Self-rated health; self-reported voter turnout to Canadian federal and municipal elections (data from Canada General Survey 2013)	Socio-demographic, socio-economic, and social capital data
Denny and Doyle (2007) [17]	Ireland	Association between health and voting	Positive significant association between subjective health and likelihood to vote in the Irish general election: an individual who reports bad health is 6.7% less likely to vote No association between psychological well-being and voter turnout	Subjective health and mental well-being (WHO-5); 2002 General Ireland election (data from European Social Survey 2005)	Education, sex, age, union membership, political ideology, income, father’s education
Denny and Doyle (2007) [18]	Britain	Association between health and voting	Positive association between voting in the general election and general health and mental health in Britain between 1979–1997 Negative association between smoking and voting Individuals with poor health are 4% less likely to vote in the 1979 and 1997 elections Smokers are 4% less likely to vote in 1979 and 1997 and 3% less likely to vote in the 1987 election compared to non-smokers.	Self-rated general health, the Malaise Inventory score and indicators of smoking and alcohol consumption; self-reported voter turnout in the 1979, 1987, and 1997 general UK elections	Sex, education, marital status, children, employment, family social class.
Habibov and Weaver (2014) [19]	Canada	Association between health and voting	Positive significant association between social capital and self-rated health in Canada Positive association of voting at all levels and self-rated health in Canada (largest positive effect on self-rated health among all of the social capital variables analyzed)	Self-rated health; self-reported voting at local, provincial, or federal level of Canadian government (data from Canada General Survey 2008)	Various sociodemographics such as age, sex, marital status, level of education, and income
Islam et al. (2006) [20]	Sweden	Association between health and voting	Positive association between municipal-level social capital (measured as voting) and better health in Sweden A municipality with a voting turnout rate 10% higher (compared to the mean election participation rate) is associated with a 2.4% higher health state score.	Generic health-related quality of life measure (HRQoL); rates of voting participation in municipal political elections (data from Statistic Sweden’s Survey of Living Condition)	Income, gender, immigration, cohabitation, education, employment, age
Islam et al. (2008) [21]	Sweden	Association between health and voting	Reduced individual risk from all-cause mortality for males 65+ who registered for municipal election participation Higher voting rate negatively and significantly associated with the mortality risk from cancer for males (*p* = 0.007), and protective associations for cardiovascular mortality (*p* = 0.134) and deaths due to “other external causes” (*p* = 0.055) Association did not hold for females.	Survival time in years and survival status at the end of follow-up period; registered Swedish municipal election participation	Income inequality, initial health status, age, income, education
Iversen (2008) [22]	Norway	Association between health and voting	Positive association of voting in municipal elections and self-assessed health in Norway The association is of considerable magnitude.	Self-assessed general health and self-assessed mental health; number of votes as a proportion of the number entitled to vote in the Norwegian local elections (data from standard-of-living survey by Statistics Norway and other sources)	Income, education
Kim and Kawachi (2006) [23]	USA	Association between health voting	Positive association between presidential electoral participation and health in the USA Those who had high social trust and electoral political participation had significantly lower odds of fair/poor health (OR = 0.56, 95% CI = 0.52–0.62; and OR = 0.78, 95% CI = 0.71–0.86, respectively).	Self-rated health; self-reported voting in the 1996 presidential election and being currently registered to vote (data from Social Capital Benchmark Study)	Age, gender, race/ethnicity, marital status, education, income, and social capital characteristics
Kim, Kim, and You (2015) [24]	OECD countries	Association between health and voting	Significant positive association between voting in the parliamentary election and subjective health controlling for sociodemographic factors Negative association between non-conventional political participation and health	Self-rated health; self-reported voting in parliamentary elections in 44 OECD countries globally (data from World Value Survey)	Age, sex, marital status, education, and income
Lahtinen et al. (2017) [25]	Finland	Association between health and voting	Results show that health exerts independent effects on voting turnout in the Finnish parliamentary, presidential, and municipal elections. Income partially mediates the effects of social capital on voting.	Use of healthcare services (including hospitalization data) and medicine purchases; individual-level register the 1999 Finnish parliamentary election and the 2012 presidential and municipal election (data from Statistics Finland)	Income, social class, age, gender, living with a partner, native language, and education
Mattila et al. (2013) [26]	Europe	Association between health and voting	Significant positive association between health and voter turnout in the European parliamentary elections, with effect most notable in older people The difference in voting probability between respondents with very good health and very bad health is 10%. The impact of health is partially mediated by social connectedness.	Self-rated health; self-reported voting in the last parliamentary election (data from European Social Survey)	One model accounted for age, gender, and education
Reitan (2003) [27]	Russia	Association between health and voting	Positive association between voter turnout in the Russian elections and life expectancy in Russia for both sexes (studied elections from 1991–1999) Overall, correlations were positive and significant.	Regional data on life expectancy (State Committee of the Russian Federation on Statistic); data on voter turnout collected from the Centre for Russian Studies at the Norwegian Institute of International Affairs (NUPI)	Unclear
Agran, MacLean, and Kitchen (2016) [28]	Western USA	Differences in voting associated with health	Qualitative article focused on lower voting rates in individuals with intellectual disabilities, and barriers and supports needed to support this community Results indicated that people with ID are interested in voting but do not receive education on political issues or voting-related decisions.	Not applicable	Not applicable
Ard et al.(2016) [29]	USA	Differences in voting associated with health	Significant positive association between engagement in politics (including voting in any US election) and self-rated health in connection to racial health disparities in the USA Social capital mediates racial disparities in health more than industrial air pollution.	Self-rated health; composite measure of electoral participation which included whether the respondent voted in the past election and is currently registered to vote (data from 2000 Social Capital Benchmark Study).	Age, sex, region of residence, marital status, and educational attainment
Bazargan, Kang, and Bazargan (1991) [7]	USA	Differences in voting associated with health	Positive association of self-rated health and voting in the US presidential election in elderly Caucasian populations: elderly Caucasians who report poor health are 13.1% less likely to vote than those reporting excellent health Positive association of life satisfaction and voting in elderly African American populations	Self-reported health; self-reported voting turnout from the US presidential election of 1980	Income, education, age, gender, living arrangement, marital status, club participation, volunteer work, health status, life satisfaction, transportation, fear of crime, union membership, demand on resources, political efficacy, political philosophy
Bazargan, Barbe, and Torres-Gil (1992) [8]	New Orleans, USA	Differences in voting associated with health	Positive association between self-rated health and voting for elderly black populations in the US elections: self-reported health status was significantly negatively associated with the number of elections voted in in the bivariate analysis, but not significant in multivariate regression analysis	Self-rated health; self-reported voting in seven elections included presidential, gubernatorial, senatorial, congressional, mayoral elections, and two propositional elections	Age, gender, education, income, accessibility of transportation, church participation, volunteer work, club participation, sense of external efficacy, sense of citizen duty, attention to public affairs, perceived difference between parties, strength of party identification
Bergstresser, Brown, and Colesante (2013) [30]	New York City, USA	Differences in voting associated with health	Qualitative study on the power of voting, social recovery, and inclusion for those with mental health issues	Not applicable	Not applicable
Gollust and Rahn (2015) [31]	USA	Differences in voting associated with health	Significant negative association between voting and those with heart disease and disabled populations in the 2008 US presidential election Significant positive association between voting, emotional support, and those with cancer	Self-reporting of chronic health condition, including diabetes, arthritis, angina/coronary heart disease), asthma, and cancer; self-reported voting in the last US presidential election (data from 2009 Behavioral Risk Factor Surveillance Survey)	Sociodemographic characteristics (age, gender, race, income, education, urbanicity) and health-related confounding factors (health insurance, disability, emotional support)
Kawachi et al. (1999) [32]	USA	Differences in voting associated with health	Negative association between female voting rate and female mortality rate: higher political participation was correlated with lower female mortality rates (*r* = − 0.51) In regression analysis, a one-unit improvement in political participation was associated with 7.3 fewer deaths per 100,000 women (95% confidence interval, CI: 3.8 to 10.9).	Total female and male mortality rates, female cause-specific death rates and mean days of activity limitations reported by women during the previous month (data from CDC); voter registration (percent women registered to vote in 1992/94), voter turnout (percent women who voted in 1992/94)	Income distribution (using the adjusted Gini coefficients), median income and poverty rates
Matsubayashi and Ueda (2014) [33]	USA	Differences in voting associated with health	Negative association between voting in the US presidential election and adults with disabilities compared to population without disabilities The odds of voting in the presidential elections from 1980 to 2008 are 50–60% lower if the respondents have work-preventing disabilities, taking into account socioeconomic factors.	Self-reported work preventing disabilities; self-reported voting rates (data from Current Population Survey)	Education and income, age, gender, and race and ethnicity
Mattila and Papageorgiou (2017) [34]	Europe	Differences in voting associated with health	Negative association between voting in the European national elections and disability; perception of discrimination increases this trend The probability of a non-disabled person voting is 80%, while the corresponding probability for those with disability and discrimination experiences is 75% (*p* < 0.01).	Disability status and disability discrimination; self-reported voting (data from European Social Survey 2012).	Age, gender, education, social connectedness
Mino et al. (2011) [35]	New York City, USA	Differences in voting associated with health	Negative association between being registered to vote in the US elections (all levels) and drug paraphernalia sharing In bivariate analysis, those registered to vote were less likely to share drug paraphernalia (33% vs. 49%; *p* = 0.046). This significance decreased in multivariate analysis, where political party identification was associated with lower drug paraphernalia sharing (adjusted odds ratio (AOR) = 0.363, CI = 0.155–0.854; *p* = 0.020).	Injection drug use health variables (sharing paraphernalia, using shooting galleries) in past 30 days; self-reported voter registration, identifying as political/part of an organized political party and attention paid to politics	All regression models controlled for age, gender, and educational level
Ojeda (2015) [36]	USA	Differences in voting associated with health	Negative association between depression and political participation (measured as voting in the US presidential election) Respondents who report no depressed mood have a 0.75 probability of voting, while respondents who report the most severe depressed mood have a probability of voting of < 0.5.	Self-reported mental health status including Center for Epidemiologic Studies Depression Scale (CES-D); self-reported voter turnout in the 1996 and 2000 US presidential elections (data from 1998 General Social Survey and the National Longitudinal Study of Adolescent Health)	Sex, race, education, age, general health, parental income, education, civic engagement, general health, marital status, church attendance, self-reported happiness (depending on data used)
Shields, Schriner, and Schriner (1998) [37]	USA	Differences in voting associated with health	Negative association between voter registration/voting rates in the 1994 US mid-term election and people with disabilities Among non-disabled respondents, 54% reported voting, while 33.1% of the people with disabilities reported voting.	Self-reported disability causing lack of work participation; self-reported registered and voted, were registered but did not vote, and voted absentee in the 1994 mid-term election (data from 1994 Current Population Survey)	Education, income, age, years of living in the community, and marital status
Sund et al. (2017) [38]	Finland	Differences in voting associated with health	Association between chronic diseases and voting in the Finnish parliamentary elections: neurodegenerative brain diseases (dementia OR = 0.20, 95% CI 0.18 to 0.22), alcoholism (OR = 0.66), and mental disorders (depression OR = 0.91; psychotic mental disease OR = 0.79) had a significant negative association, whereas cancer and COPD/asthma had a positive association (both OR = 1.05). Having more than one condition further decreased voting probability (1 condition OR = 0.96, 2 conditions OR = 0.83, 3 conditions OR = 0.68 and 4+ conditions OR = 0.50)	Hospital discharge diagnoses and reimbursements for drugs prescribed, to identify persons with 17 chronic hospital-treated diseases; individual-level register records for the 1999 Finnish parliamentary elections	Gender, age, education, occupational class, income, partnership status, cohabitation with underaged children and hospitalization during election day
Urbatsch (2017) [39]	Finland, USA	Differences in voting associated with health	Association between low voter turnout and influenza outbreaks in USA and Finland In Finland, influenza prevalence reduces turnout in Finnish residential, parliamentary, and municipal elections by 2.1% (95% CI: 21.2 to 23.1 percentage points). In the USA, a higher level of influenza reduces turnout in the US presidential and state elections by 1.2% (95% CI: 20.4 to 22.1).	Influenza infections; voter turnout is measured as a share of the voting-eligible population at major elections (statistics from the national Finnish and US surveillance systems)	Healthcare access, population > 65, population per square meter, type of election
Rodriguez (2018) [40]	USA	Electoral implications	Positive association between health and political participation causes early mortality of poor people. Health differences between 10-year survivors and non-survivors explain 56% of their differences in socio-political participation. Without detrimental differences in health, individuals would participate 28% more as they age. High-SES survivors participate 60% more than low-SES survivors and 85% more than low-SES non-survivors.	Mortality status and self-rated health; index of political participation (volunteering, attending meetings, and giving money) (data from Midlife in the United States: a national study of health and well-being)	Education, income
Rodriguez et al. (2015) [41]	USA	Electoral implications	Excess mortality in African American populations from 1970 to 2004 (2.7 million deaths) due to health inequality affected 2004 US presidential and state election outcomes (1 million lost black votes)	Deaths by state (data from Multiple Cause of Death files 1970–2004); total number of votes by state (data from US Elections Project, National Election Pool General Election Exit Polls (2004).	Sex, race, age, region
Ziegenfuss, Davern and Blewett (2008) [42]	USA	Electoral implications	Comparison of proportion of those who delayed accessing health care and voted in 2004 compared with the 2000 US national election Those who delay healthcare care were less likely to vote than those who did not in 2000, but not in 2004. In 2004, those who delayed care and voted were more than twice as likely to vote Democratic than Republican.	Access to healthcare; self-reported voting in the 2000 and 2004 presidential elections (data from American National Election Study)	Age, gender, race/ethnicity, income, marital status, educational attainment, party identification, home ownership, church attendance, and length of time residing in the same home or apartment
Anderson and Dabelko-Schoeny (2010) [43]	USA	Healthcare interventions	Commentary on civic engagement leading to better health in nursing home residents from social worker perspective, with call to action for social works to engage	Not applicable	Not applicable
Hassell and Settle (2017) [44]	USA	Healthcare interventions	Study experimented with interventions on life stress and likelihood to vote in the US presidential and municipal elections When triggered with life stressors, individuals without a history of voting were significantly less likely to vote while routine voters were unaffected. Non-voters exposed to the life stressors reduced likelihood of voting by 5%.	Life stressors; self-reported voting in the 2012 US presidential election and the 2013 municipal election in a small Midwestern American town	Used control groups in field experiments
Liggett et al. (2014) [45]	Bronx, USA	Healthcare interventions	Study examined a clinician-led voter registration drive within 2 university-affiliated health centers in the Bronx, New York. 38% of the total patients engaged in voter registration drive were registered to vote for the 2008 US presidential election: 114 of the 304 patients engaged were registered, of which 54% were first-time registrants	Not applicable	Not applicable
Regan, Hudson, and McRory (2011) [46]	USA	Healthcare interventions	Literature review of patient participation in public elections, with call to action for nurses to engage in promoting patients’ right to vote through policy guidelines and a flexible and proactive nursing approach to participation	Not applicable	Not applicable
Wass et al, (2017) [47]	Europe	Healthcare interventions	Voter facilitation instruments (advance/postal voting, voting outside the polling stations) for parliamentary elections in 30 European countries have insignificant effects to increase electoral participation for those suffering from ill health or disabilities (except proxy voting)	Self-rated disability and self-rated health; self-reported voter turnout (data from European Social Survey)	Gender, age, education and cohabitation with a spouse
White and Wyrko (2011) [48]	UK	Healthcare interventions	Commentary encouraging voter outreach in the UK elections to older patients admitted to geriatric rehab hospital	Not applicable	Not applicable

Four common themes emerged: (1) there are consistent patterns in the association between voting and health; (2) differences in voter participation are associated with health conditions; (3) gaps in voter participation may be related to electoral outcomes; and (4) healthcare interventions exist to increase voting and democratic engagement. We chose these four overarching themes after reviewing summarized notes of the key findings and details of each included article (see Methods). Although there is partial overlap, we believe that articles included under each theme deliver four distinct messages that inform our main research questions in unique ways.

### Consistent patterns in the association between voting and health

Seventeen studies examined the association between voting and health in numerous jurisdictions and levels of government (municipal, state or province, and federal elections), and in numerous locations across North America and Europe. Lower voting rates are consistently associated with poor self-rated health. In most studies, health was measured by surveys that included questions about self-reported health [6, 13, 16–19, 22–24, 26]. Other measures included health risk behaviors [12, 14], mortality [21, 27], chronic health conditions [14], health indices [14, 15, 18, 20], and hospitalization data [25]. This health data was then linked to data on voting, measured in various ways including self-reported voting registration and national statistics. Blakely, Kennedy, and Kawachi analyzed the data of 280,000 respondents of an American Current Population Survey and found that voting is positively associated with self-rated health, independent of income inequality [6]. Similar patterns were found by Burden et al. in an older Wisconsin population [15]. Globally, similar correlations between voting and health have been found in Ireland [18], Russia [27], Sweden [20], Canada [16, 19], Europe [26], and the OECD more broadly [24]. Both Couture and Breux [16] and Habibov and Weaver [19] looked at large sample sizes from Canada’s General Social Survey and found a correlation between self-rated health and voting. Habibov and Weaver connected this association between voting and health to the importance of social capital [19], as did many other articles in our review [6, 21–23, 26, 29, 30, 38].

Most studies were cross-sectional, with only a few longitudinal studies finding an association between voting and health and socioeconomic benefits. Adjusting for confounders like sex, education, geography, and chronic illness, Arath showed that voting abstention was associated with 1.3 times higher odds of reporting poor health two years later [13]. Ballard, Hoyt, and Pachucki looked at longitudinal data that followed adolescents into adulthood and found that voting was positively associated with better mental health and health behaviors over time, along with improved income and education level [14].

### Differences in voter participation are associated with health conditions

Although the connection between voting and health was researched in the above articles, the next overarching theme further analyzes this connection by discussing voting patterns in distinct sub-populations. People with physical, intellectual, and psychological disabilities have lower rates of voting. Agran, MacLean, and Kitchen found lower voting rates in communities of people with intellectual disabilities [28]. Matsubayashi and Ueda [33], Mattila and Papageorgiou [34], and Shields, Schriner, and Schriner [37] discovered low voter turnout rates among people with disabilities, with barriers to voting including discrimination and accessibility. Mental health and addiction can also impact voting. Mino et al. found a negative association between being registered to vote and harmful drug injection behavior (ex. sharing paraphernalia) [35], and Ojeda found that depression reduced voting participation [36]. In a qualitative study, Bergstresser, Brown, and Colesante interviewed 52 consumers of mental health services who described political participation as contributing to their recovery by increasing social inclusion [30].

There are differences in voter participation by race, gender, age, and disease type. Ard et al. found a positive association between engagement in politics and self-rated health in connection to racial health disparities in the USA [29]. Disparities in health and voting in African American communities were found in two studies by Bazargan, Kang, and Bazargan [7] and Bazargan, Barbre, and Torres-Gil [8], which saw a voting gap in elderly black communities in the USA. Being elderly can lead to certain vulnerabilities, such as social isolation and physical impairment, which can then lead to lower voting rates [26, 43]. Higher political participation (which includes voting participation and registration to vote) in American women is also strongly correlated with lower mortality [32]. Interestingly, the type of disease an individual has can affect their voting behavior. Acute illnesses like influenza can affect voter turnout [39]. Focusing primarily on chronic diseases, Gollust and Rahn found that those with heart disease and disability were less likely to vote in the 2008 US election, whereas those with cancer were more likely to vote [31]. One hypothesis was that strong social support networks in the cancer community, and less stigma compared to other diseases, led to higher voting rates among people with cancer. Sund et al. saw similar results: those with cancer and COPD often voted more, whereas those with neurodegenerative brain disease, addiction, and mental health disorders voted less [38].

### Gaps in voter participation may be related to electoral outcomes

Although only three articles were included under this theme, we nonetheless created a distinct category due to the unique and important findings of these articles, namely, differences in health status and subsequent differences in voting patterns can impact electoral outcomes. In two population health studies, Rodriguez [40] and Rodriguez et al. [41] analyzed the association between poor health and voting and the broader impact these inequities can have on our political systems. They hypothesize that “through the early disappearance (i.e., death) of the poor, continuing socio-political participation of high-SES survivors helps to perpetuate inequality in the status quo” [40]. The citizens most expected to vote in line with redistributive health policies are the same citizens that have higher mortality rates during the time when they are most likely to vote—middle age. Previous to this study, Rodriguez et al. looked at how racial inequality in the USA leads to excess mortality and therefore a loss of votes. In introducing the subject of racism and voting, Rodriguez et al. point out current US voter suppression practices aimed at marginalizing minority populations, from felony disenfranchisement laws, to redrawing of electoral boundaries, to shortened polling hours. This article focuses on the effects of health inequity as another threat to minority voting power. They found that from 1970–2004, there were 2.7 million excess black deaths due to racial inequality, which led to 1 million lost black votes in the 2004 election [41]. This study concluded that many close state-level elections in the US over this period of time would likely have had different electoral outcomes if not for these excess mortality rates.

Using a multivariate analysis and controlling for sociodemographic characteristics, Ziegenfuss, Davern, and Blewett [42] found that individuals with healthcare access problems were significantly more likely to vote for Democratic candidates in the 2004 election. They connected this to the Democratic Party comprehensive approach to healthcare reform in the 2004 election. If inequities in access to healthcare services and in health outcomes can change who wins elections, a vicious cycle can emerge: worse health leads to lower voting rates, leading to policy that does not prioritize addressing inequities, leading to worsening health inequities.

### Healthcare interventions exist to increase voting and democratic engagement

Healthcare interventions aimed at increasing voting rates have emerged within nursing, social work, and medicine. Regan, Hudson, and McRory conducted a literature review that looked at the role of nurses in ensuring patients’ right to vote, issuing a call to action for nurses to help ensure this right through policy guidelines and increased support for patients [46]. Anderson and Dabelko-Schoeny argued that civic engagement can lead to better health in nursing home residents and called for social workers to develop and implement interventions that increase engagement [43]. White and Wyrko wrote that healthcare professionals should make every effort to ensure hospital patients can vote in the UK [48]. They suggest an approach focused on increased awareness and discussion among healthcare practitioners, promotion of voting access, and the consideration of emergency proxy voting.

Within the healthcare setting, Wass et al. found that proxy voting as a voter facilitator instrument can increase voter turnout for those suffering from ill health or disability [47]. Hassell and Settle ran an interventional study that induced life stressors on patients and found that increasing stress decreased likelihood to vote for typical non-voters [44]. Liggett et al. conducted an evaluation of clinician-led, nonpartisan voter registration drives over 12 weeks within two university-affiliated health centers in the Bronx, New York [45]. The project was successful in registering 89% of eligible voters, demonstrating the importance of health centers as, “powerful vehicles for bringing a voice to civically disenfranchised communities”.

## Discussion

Our review found an association between voting and health. Poor health is often associated with lower rates of voting. This was consistent across diverse health outcomes, jurisdictions and governments. A few studies provided weak evidence that voting may lead to better health and well-being [13, 14], although there have not been enough studies in this area to strongly confirm this association. Individuals living with disability, mental and physical illness, minorities, and older individuals, tend to vote at lower rates in general. Votes lost to morbidity and mortality in marginalized populations may potentially impact electoral and policy outcomes, including public health policy. Among some of the included studies, the causal relationship between voting and health was seen as bidirectional: voting affects health as it shapes who is in power and what policy is made, and individual health can affect voting. Taken together, a cycle can develop of poor health and political disempowerment, although further research is required to fully characterize this process. Despite the importance of this relationship, the association between voting and health has not received significant attention in the public health literature to date [49]. This review provides some conceptual clarity to this developing research area.

Many articles included calls to action for healthcare practitioners to engage in and advocate for democratic engagement in their patient communities through policy change, accessibility, support, and even intervention to help increase voter participation. Healthcare organizations are well suited to engage directly with marginalized populations and can be involved in improving democratic engagement through education and interventions similar to Liggett et al., who undertook a clinician-led voter registration [45]. Other possible interventions could include reducing barriers to voting (proxy voting at hospitals), organizing nonpartisan townhalls, or compiling and sharing information for communities on the voting process [50, 51].

Many authors proposed theories to explain why poor health and lower voting turnout were associated. These included that people with poor health had lower cognitive resources, worse sense of efficacy, unmet accessibility needs (especially for those with disabilities), and limitations in time, social/emotional, and financial resources due to health burden [25, 29, 31]. Several authors cited social capital and social connectedness as part of the causal link between voting and health. Voting could be seen as a form of social capital as it entails social trust and civic engagement, but even more than that, having social networks who vote and talk about voting can reinforce voting patterns within a community. Social connectedness can improve mental and physical health, lead to less risky health behaviors, and increase access to community networks, institutions, and resources to improve health [16, 20–22]. Gollust and Rahn explored the role social capital played in voting and health by discussing one of the only populations where voting rates increase with a chronic health condition: people living with cancer [31]. They hypothesized that people living with cancer are much more likely to join social and advocacy cancer support groups than people with other diseases. For example, people with breast cancer form more than forty times more support groups than people with heart disease. These social and advocacy groups not only then support the act of voting, but also equip members with skills that help them better understand the political process, which then leads to higher voter participation. Overall, many authors linked voting to health through social capital. This is an important area of future research for the field of public health, as social capital is a key social determinant of health in itself [3]. This links back to an important consideration in our scoping review: how voting is connected to the social determinants of health.

Our review had limitations. Voting was chosen as a proxy for democratic engagement, but there are numerous other forms of democratic engagement: activism, protest, donations to political groups, political education, and more. Also, democracy comes in many forms, between countries and within countries at different times. We recognize this would influence how voting occurs in different contexts, the meaning it would have to citizens, and the subsequent relationship between voting and health. Voting is also deeply connected with other social determinants of health—namely income and education—which may confound some of the research presented. Most of the articles addressed confounders within their statistical analysis, including sex, age, marital status, race, education, employment, income, geography, and more. Addressing these confounders was imperative in claiming an association between voting and health, but it is important to note that the articles often measured these factors differently and used a differing combination of factors. Future work should synthesize this literature to develop a more holistic picture of the connection between other forms of democratic engagement and health. Future research should also examine the long-term effects of voting on health, as well as the impact of health organizations actively intervening in and advocating for democratic engagement in their communities.

## Conclusion

This review has supported the association between voting and health. Communities marginalized by disability, mental and physical health, race, and age tend to be the most affected by the positive association between health and voting. Differences in voter participation related to health inequities can have some effect on overall electoral outcomes, shaping overall policy and possibly deepening healthcare inequities. Future research should study the long-term effects of voting on health, the effects of other forms of democratic engagement on health, and the impact healthcare practitioners can have on voting activity in their community through intervention and advocacy.

## Supplementary information

**Additional file 1:** MEDLINE search strategy.

**Additional file 2:** Articles identified by database.

## Data Availability

Not applicable
