# Supplementary material for: Voting, health and interventions in healthcare settings: a scoping review
Source: Public Health Rev. 2020 Jul 1;41:16. doi: 10.1186/s40985-020-00133-6 (PMC7329475; doi:10.1186/s40985-020-00133-6)
Supplement: Supplementary file 1 — Additional file 1: MEDLINE search strategy. [file 40985_2020_133_MOESM1_ESM.docx]

**Additional file 1: MEDLINE search strategy**

1 (politic* adj3 (participat* or engage*)).ti. (147)

2 (democratic adj3 (participat* or engage*)).tw,kf. (99)

3 (civic adj3 (participat* or engage*)).ti. (108)

4 ((electoral or election*) adj3 (participat* or engage*)).tw,kf. (48)

5 (vote* or voting).ti. (1783)

6 activis*.ti. (1219)

7 1 or 2 or 3 or 4 or 5 or 6 (3373)

8 exp Health Status/ (280725)

9 health*.tw,kf. (2304405)

10 8 or 9 (2448504)

11 7 and 10 (654)

12 limit 11 to english language (605)
