# Supplementary material for: Voting, health and interventions in healthcare settings: a scoping review
Source: Public Health Rev. 2020 Jul 1;41:16. doi: 10.1186/s40985-020-00133-6 (PMC7329475; doi:10.1186/s40985-020-00133-6)
Supplement: Supplementary file 2 — Additional file 2: Articles identified by database. [file 40985_2020_133_MOESM2_ESM.docx]

**Additional file 2: Articles identified by database**

| **Databases searched** | **Date of search** | **Number of results** |
| --- | --- | --- |
| All Ovid Medline <1946 - present> | March 8, 2018 | 605 |
| PsycINFO <1806 to February Week 4 2018> | March 8, 2018 | 709 |
| Ebsco CINAHL | March 9, 2018 | 248 |
| Embase Classic+Embase <1947 to 2018 March 08> | March 9, 2018 | 609 |
| Web of Science (including Social Sciences Citation Index) | March 9, 2018 | 679 |
| ProQuest Sociological Abstracts | March 9, 2018 | 864 |
| ProQuest Worldwide Political Science Abstracts | March 12, 2018 | 114 |
| Total | 2965 |  |
| **Total**  **(after deduplication)** | **2041** |  |
